# Long-term clinical and echocardiographic outcomes after mitral valve surgery for severe mitral annular calcification

**DOI:** 10.3389/fcvm.2026.1881651

**Published:** 2026-10-28

**Authors:** Yoganiranjana Dharuman, Egan Kalmykov, Mirko Doss

**Affiliations:** 1 Medical Faculty, RWTH Aachen University, Aachen, Germany; 2 Department of Endovascular Surgery, University Clinic of Brandenburg/Havel, Brandenburg, Germany; 3 Department of Cardiothoracic Surgery, Helios Hospital Siegburg, Siegburg, Germany

**Keywords:** decalcification, mitral annular calcification, mitral valve repair, mitral valve surgery, patch reconstruction

## Abstract

**Objective:**

To assess the long-term clinical and echocardiographic outcomes of mitral valve surgery with annular decalcification and pericardial patch reconstruction in patients with severe mitral annular calcification (MAC).

**Patients and methods:**

Between 2012 and 2022, 112 consecutive high-risk patients (67 women, 45 men; mean age 66.5 ± 13.6 years) underwent surgery for severe MAC with mitral stenosis and/or regurgitation. Mitral valve repair was performed in 55 patients (49%) and replacement in 57 (51%). Concomitant procedures were performed in 65 patients (58%). Mean follow-up was 97 ± 48 months.

**Results:**

Early mortality was 6.3% (*n* = 7) and late mortality was 23.2% (*n* = 26). Survival was 89.3%, 77.2%, and 64.1% at 60, 96, and 144 months, respectively. Freedom from reoperation was 98.3% at 60 months and 93.3% at 96 months. At follow-up, no surviving patient had more than grade III mitral regurgitation. Grade III regurgitation was present in 3 patients (4%), while no patient had grade IV regurgitation. Left atrial diameter, left ventricular end-diastolic diameter, and mean transvalvular gradient were significantly lower than before surgery.

**Conclusion:**

In this high-risk cohort, annular decalcification and pericardial patch reconstruction provided durable valve function and acceptable long-term survival in patients who survived the perioperative period. Perioperative mortality, however, remained substantial.

## Introduction

An increasing number of elderly patients undergo cardiac surgery, and many carry several comorbidities that raise the perioperative risk (1). MAC becomes more frequent with age and is more common in women (2); in the Framingham cohort its prevalence was 2.8% (3).

Severe annular calcification makes mitral valve surgery technically demanding. Dense calcium hinders secure suture placement and stable seating of a prosthesis and increases the risk of atrioventricular disruption, left ventricular rupture and paravalvular leakage. The extent of calcification and the feasibility of repair therefore have to be assessed before and during the operation (4). In addition, extensive annular calcification may limit the available surgical options and requires careful consideration of the relationship between the calcified annulus, the mitral valve apparatus and the surrounding cardiac structures. The surgical strategy must therefore be individualized according to the distribution and extent of calcification, the underlying valve pathology and the feasibility of achieving a durable repair.

At our institution, severe MAC was treated by en-bloc removal of the calcified bar, reconstruction of the annulus with a pericardial patch and, where feasible, valve repair rather than replacement (5, 6). This approach aims to restore an adequate annular structure while allowing reconstruction of the mitral valve in patients in whom conventional techniques may be particularly challenging. Extensive decalcification and annular reconstruction, however, represent technically demanding procedures that require meticulous surgical handling and careful assessment of the reconstructed annulus and valve.

Long-term clinical and echocardiographic data after extensive annular decalcification and reconstruction are limited. In particular, the durability of the reconstructed annulus, the subsequent function of the mitral valve and the occurrence of late complications remain important considerations. Evaluation of both clinical outcomes and echocardiographic findings is therefore essential to assess the long-term results of this surgical strategy.

We therefore reviewed our consecutive experience, with attention to early and late mortality, perioperative morbidity, valve function and survival. The aim was to characterize the early and long-term outcomes of patients undergoing extensive annular decalcification and reconstruction for severe MAC and to assess the durability of the surgical approach over extended follow-up.

### Patients and methods

Between 2012 and 2022, 112 consecutive patients with haemodynamically significant mitral valve disease (mitral stenosis and/or regurgitation) and severe MAC underwent surgery in our department. Surgery was limited to the mitral valve in 47 patients (42%); 65 patients (58%) underwent one or more additional procedures (Table 1). Follow-up was updated in 2024 and was complete for 98.4% of the cohort.

**Table 1:** Concomitant procedures performed in addition to mitral valve replacement or repair.

Concomitant procedure	Number (n)	Percentage (%)
Myocardial revascularization (CABG)	33	29.5%
Aortic valve replacement (AVR)	21	19.4%
Aortic valve repair (AVr)	3	2.6%
Tricuspid valve repair (TVR)	23	20.4%
Plication of the ascending aorta	5	4.3%
Atrial ablation	31	28.6%

Percentages are of the total cohort (*n* = 112). Some patients underwent more than one concomitant procedure.

Patients with only mild or moderate annular calcification were excluded, as were those in whom MAC was not associated with haemodynamically significant mitral valve disease. Further exclusion criteria were age below 18 years, transcatheter mitral procedures and emergency or salvage operations.

Fifty-seven patients (51%) underwent replacement (biological *n* = 38, mechanical *n* = 19) and 55 (49%) underwent repair. All patients had preoperative coronary angiography, which showed coronary artery disease requiring treatment in 33 patients (29%). Eighty patients (71%) were in NYHA class III or IV and 32 (29%) in class I or II. Mean predicted operative mortality (EuroSCORE II) was 18.8% (7). Echocardiography showed involvement of at least one third of the posterior annulus, in most cases extending to a commissure and the anterior annulus, in keeping with the Carpentier classification (5). Baseline demographic and echocardiographic data are shown in Table 2.

**Table 2:** Preoperative demographic and echocardiographic patient data (*n* = 112).

Variable	Value
Demographics	
Total number (n)	112
Female	67 (60%)
Male	45 (40%)
Age, years (mean ± SD)	66.5 ± 13.6
Female	70.3 ± 11.7
Male	60.7 ± 14.3
Operative risk	
Mean EuroSCORE II	18.8%
Valve pathology	
Mitral stenosis	28 (25%)
Mean transvalvular pressure gradient	23 ± 3.2 mmHg
Mean mitral valve area	0.76 ± 0.4 cm^2^
Mitral regurgitation	83 (75%)
Moderate to severe mitral regurgitation	74 (66%)
Etiology of dysfunction	
Degenerative changes	93 (84%)
Barlow's disease	11 (10%)
Endocarditis	5 (4%)
Rheumatic heart disease	2 (2%)
Cardiac function & dimensions	
Mean left ventricular ejection fraction	56 ± 15%
Mean left ventricular end-diastolic diameter	57 ± 16 mm
Mean left atrial diameter	54 ± 7 mm
Risk factors & comorbidities	
Arterial hypertension	35 (31%)
Pulmonary hypertension	35 (32%)
Diabetes mellitus	23 (21%)
Insulin-dependent	9 (8%)
Non-insulin-dependent	14 (13%)
Hypercholesterolemia	18 (16%)
Hyperuricemia	4 (3%)
History of stroke	7 (7%)
Permanent atrial fibrillation	46 (42%)
Paroxysmal atrial fibrillation	45 (41%)
Sick sinus syndrome	2 (2%)
AV block (1st and 2nd degree)	2 (2%)
Chronic renal failure (creatinine > 200 µg/l)	16 (14%)
Dialysis dependence	8 (6%)
Previous cardiac surgery	12 (10%)
History of cardiac decompensation	18 (16%)
COPD or asthma	18 (16%)
Liver cirrhosis	3 (2%)

SD, standard deviation. EuroSCORE II is reported as predicted operative mortality.

### Surgical technique

A full median sternotomy was used in 80 patients (71%) and a partial upper sternotomy in 16 (14%). In the remaining 16 patients (14%) a minimally invasive approach was used through a 5-8 cm right anterolateral submammary incision with thoracotomy in the fourth intercostal space (Chitwood technique) (8). The approach was selected individually according to body habitus, anatomy and the need for concomitant procedures; the minimally invasive approach was reserved for isolated mitral valve disease with favourable habitus and anatomy, and full or partial sternotomy was used when concomitant procedures, redo surgery or unfavourable anatomy were present. For sternotomy and partial sternotomy, the ascending aorta and both venae cavae were cannulated; for the Chitwood technique the femoral vessels were used, the aorta was cross-clamped transthoracically through a 7 mm incision in the second or third intercostal space with a Chitwood clamp, and blood cardioplegia was delivered into the aortic root (8). Cardiopulmonary bypass was conducted under moderate hypothermia (32 °C). In the sternotomy group the mitral valve was exposed through the interatrial groove and, later in the series, transseptally; in a subset of patients exposure was improved by transecting the superior vena cava. After inspection of the leaflets and subvalvular apparatus, the choice between repair and replacement was made and the extent of calcification was assessed.

The posterior leaflet was detached from the calcified annulus and the calcium removed en bloc with scalpel and scissors. The defect in the atrioventricular junction was closed with a pericardial patch, initially untreated autologous pericardium and later bovine pericardium. When replacement was chosen, the posterior leaflet was preserved as far as possible and the mural sutures were passed through the atrial wall, the pericardial patch, any residual mural leaflet and the sewing ring.

When the valve was repaired, the sutures were passed through the atrial wall or its cut edge and tangentially through the patch; before ring implantation the remaining mural leaflet was reattached to the patch, folded into the mitral orifice, with a double continuous suture, which allowed augmentation of a mural leaflet left narrow after calcium removal. Figure 1 shows a calcium bar after decalcification.

**Figure 1:**
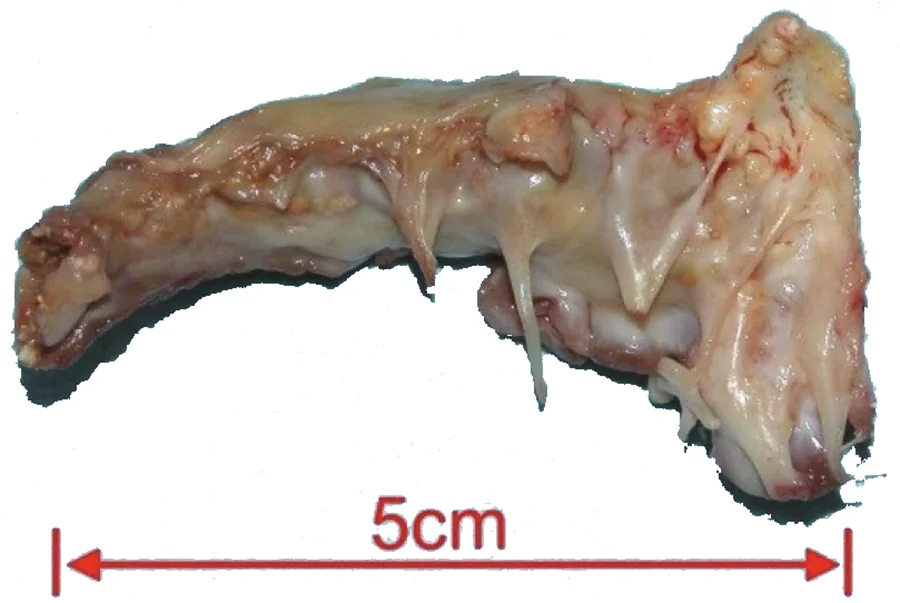
Calcified segment after decalcification of the mitral annulus.

Mean follow-up was 8 ± 4 years, with a minimum follow-up of 2 years. Early mortality was defined as death within 30 days of surgery or during the index hospital stay, perioperative mortality as death within three months, and late mortality as death more than 30 days after surgery and after discharge. Valve function was assessed by echocardiography, and symptoms, complications and exercise tolerance were recorded through a structured telephone questionnaire.

### Statistical analysis

Analyses were performed in BiAS for Windows (version 11.10; Epsilon-Verlag, Frankfurt, Germany), with data managed in Microsoft Excel 2021 (Microsoft Corporation, Redmond, WA, USA). Normality was assessed with the Shapiro–Wilk test and Q-Q plots. Normally distributed variables are given as mean ± SD, non-normally distributed variables as median (IQR) and categorical variables as n (%). Operative, bypass and cross-clamp times were compared between survivors and non-survivors with the Wilcoxon-Mann–Whitney test, and paired pre- and postoperative echocardiographic measurements with the paired Wilcoxon test. Survival and freedom from reoperation were estimated by the Kaplan–Meier method, and the association of preoperative variables with survival was tested by univariate log-rank analysis. No multivariable Cox model was fitted, and the results are therefore associations rather than independent predictors. Observed survival was compared with the expected survival of an age- and sex-matched general population by the one-sample log-rank test. A two-sided *p* < 0.05 was considered significant.

## Results

### Perioperative data

Mean operative time was 317 ± 87 min, cardiopulmonary bypass time 198 ± 61 min and aortic cross-clamp time 135 ± 44 min. For combined procedures these were 348 ± 81, 222 ± 57 and 152 ± 41 min; for isolated repair 269 ± 82, 169 ± 50 and 114 ± 31 min; and for isolated replacement 302 ± 81, 161 ± 64 and 111 ± 48 min. Mean hospital stay was 14.7 ± 9.4 days and mean intensive-care stay 4.8 ± 7.3 days.

### Peri- and postoperative complications

Complications were low cardiac output syndrome (4.5%, *n* = 5), third-degree AV block requiring a pacemaker (0.9%, *n* = 1), stroke (1.8%, *n* = 2), acute renal failure requiring dialysis (6.3%, *n* = 7), respiratory insufficiency (10.7%, *n* = 12) and gastrointestinal ulcer bleeding (7.1%, *n* = 8). Re-thoracotomy was performed for diffuse bleeding or pericardial tamponade in 8 patients (7%). Four patients (3.6%) required early reoperation for recurrent mitral regurgitation or paravalvular leakage (Table 3).

**Table 3:** Early postoperative outcomes and complications (*n* = 112).

Outcome/complication	n	%
30-day (early) mortality	7	6.3%
Intraoperative death (LV rupture/biventricular failure)	2	1.7%
Low cardiac output syndrome	3	2.5%
Stroke	2	1.8%
Third-degree AV block requiring pacemaker	1	0.9%
Acute renal failure requiring dialysis	7	6.3%
Respiratory insufficiency	12	10.7%
Gastrointestinal bleeding (ulcers)	8	7.1%
Re-thoracotomy for bleeding/tamponade	8	8%
Early reoperation (recurrent MR/paravalvular leak)	4	3.6%

LV, left ventricular; MR, mitral regurgitation. Percentages are of the total cohort (*n* = 112).

### Late complications during follow-up

Thromboembolic events occurred in 6 patients (5.4%), in most cases without residual deficit; 2 patients reported paresis with mild limitation. Gastrointestinal ulcer bleeding occurred in 4 patients (3.6%), of whom 3 were taking phenprocoumon. Late reoperation was required in 3 patients (2.7%) for endocarditis and in 2 (1.7%) for paravalvular leakage.

By the end of follow-up, 33 of the 112 patients (29.5%) had died. Early mortality was 6.3% (7 patients); 2 of these (1.7%) died intraoperatively from left ventricular rupture or biventricular failure, and the remaining early deaths were due to low cardiac output syndrome (2.7%, *n* = 3) and multi-organ failure (1.7%, *n* = 2). Late mortality was 23.2% (*n* = 26): left ventricular failure (10.7%, *n* = 12), sepsis, pneumonia or multi-organ failure (3.6%, *n* = 4), sudden cardiac death (2.7%, *n* = 3), malignancy (0.9%, *n* = 1) and refractory arrhythmia (0.9%, *n* = 1); the cause was not established in 5 patients (4.5%).

On univariate analysis, higher early mortality was associated with arterial hypertension (*p* = 0.0003), diabetes mellitus (*p* = 0.0050), age over 65 years (*p* = 0.0323), concomitant procedures (*p* = 0.0382), aortic valve replacement (*p* = 0.0047), combined replacement and a concomitant procedure (*p* = 0.0062) and previous cardiac decompensation (*p* = 0.0002).

Higher late mortality was associated with arterial hypertension (*p* = 0.001), diabetes mellitus (*p* = 0.0007), chronic renal failure (*p* = 0.0019), age over 65 years (*p* = 0.0155) and previous cardiac decompensation (*p* = 0.0001). Mortality differed by procedure. Of the 57 patients who underwent replacement, 5 (8.8%) died within 30 days and 16 (28.1%) later, an overall mortality of 36.8%. Of the 55 repair patients, 2 (3.6%) died within 30 days and 10 (18.2%) later, an overall mortality of 21.8%. Twenty-one patients underwent additional aortic valve replacement; 6 of the 7 patients who died within 30 days had undergone double-valve surgery, and concomitant aortic valve replacement was associated with early mortality (*p* = 0.0047). Survivors had shorter operative (*p* = 0.0339), bypass (*p* = 0.0139) and cross-clamp times (*p* = 0.0085) than non-survivors.

Follow-up was complete for 98.4% of the cohort. Long-term transthoracic echocardiography was available in 72 of the 79 patients alive at follow-up (91.1%). No mitral regurgitation was present in 47 patients (65.3%); 17 (23.2%) had grade I, 4 (5.6%) grade II and 3 (4%) grade III, and no patient had grade IV. Mean mitral valve area was 2.7 cm^2^, mean transvalvular gradient 5.8 mmHg, peak gradient 11.8 mmHg and mean left ventricular ejection fraction 58%. Mean left atrial diameter was 41 mm and mean LVEDD 51 mm. Compared with baseline, left atrial diameter, LVEDD and mean transvalvular gradient were significantly lower (*p* < 0.0001, *p* = 0.0035 and *p* < 0.0001, respectively) (Table 4).

**Table 4:** Long-term echocardiographic data of surviving patients (*n* = 72).

Echocardiographic parameter	Value
Residual mitral regurgitation grade	
None	47 (65.3%)
Grade I	17 (23.2%)
Grade II	4 (5.6%)
Grade III	3 (4%)
Grade IV	0 (0%)
Valve & cardiac parameters	
Mean mitral valve area	2.7 ± 0.8 cm^2^
Mean transvalvular pressure gradient	5.8 ± 1.4 mmHg
Maximum pressure gradient	11.8 ± 2.6 mmHg
Mean left ventricular ejection fraction	58 ± 8%
Mean left atrial diameter	41 ± 5 mm
Mean left ventricular end-diastolic diameter	51 ± 6 mm

Data are from 72 of the 79 surviving patients with available long-term echocardiography.

At follow-up, 48 patients (43%) were in NYHA class I, 19 (17%) in class II and 5 (4.5%) in class III, and none in class IV. Before surgery, 9 patients (8%) had been in class I, 21 (18%) in class II, 25 (22%) in class III and 17 (15%) in class IV.

Survival was 89.3%, 77.2% and 64.1% at 5, 8 and 12 years. Expected survival for an age- and sex-matched general population was taken from the period life tables of the German Federal Statistical Office (Destatis). After exclusion of perioperative (three-month) deaths, survival did not differ significantly from the matched population (*p* = 0.4301) (Figures 2, 3); this represents conditional survival among patients who survived the perioperative period and does not reflect the full surgical mortality. On the one-sample log-rank test, observed and expected deaths were 33 versus 22.2 with perioperative mortality included (*p* = 0.0016) and 24 versus 22.2 after its exclusion (*p* = 0.4301).

**Figure 2:**
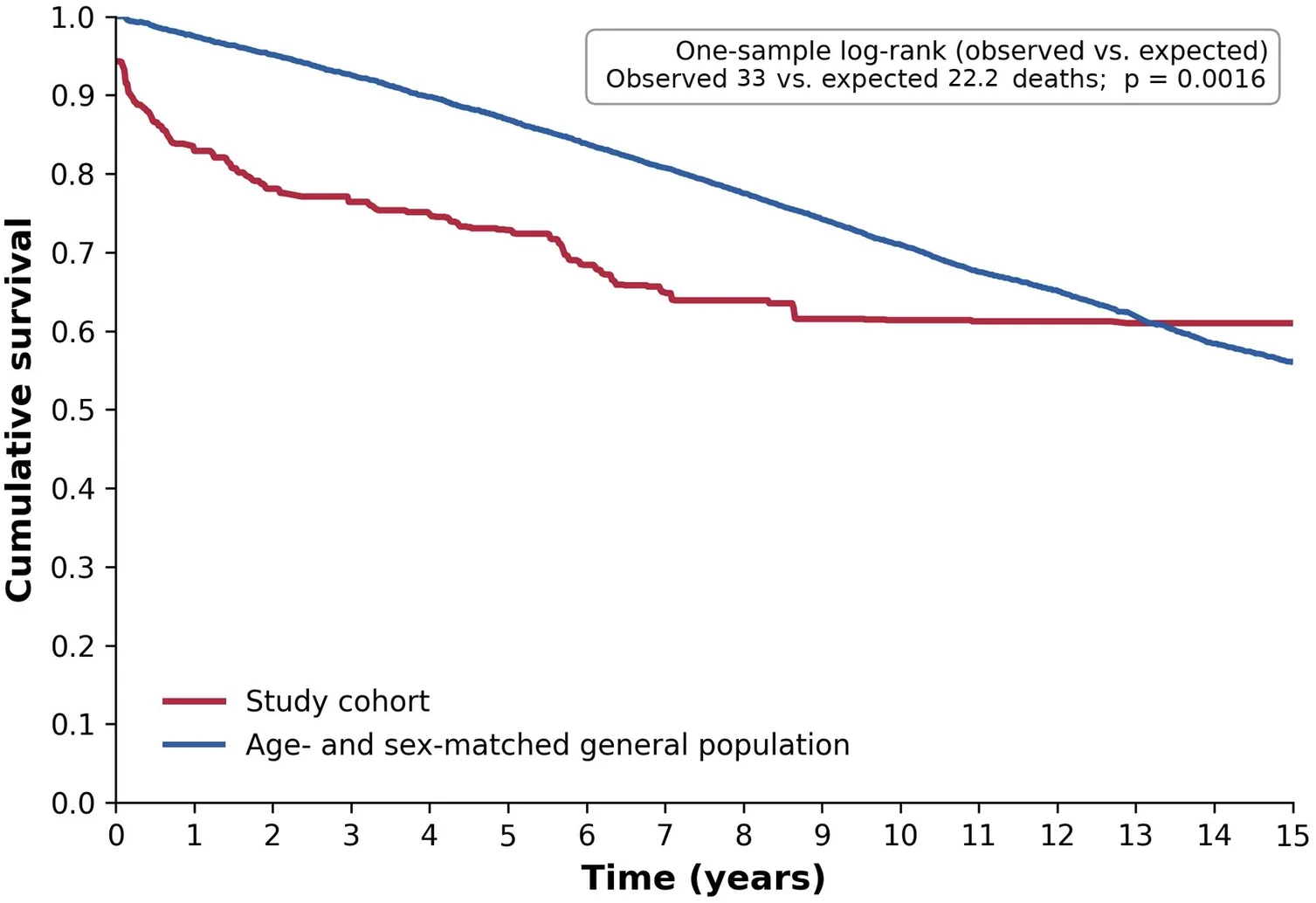
Survival comparison of the study cohort with an age- and sex-matched general population, including perioperative mortality. Red line, study cohort (Kaplan–Meier); blue line, matched general population. One-sample log-rank test (observed vs. expected): observed 33 vs. expected 22.2 deaths; *p* = 0.0016.

**Figure 3:**
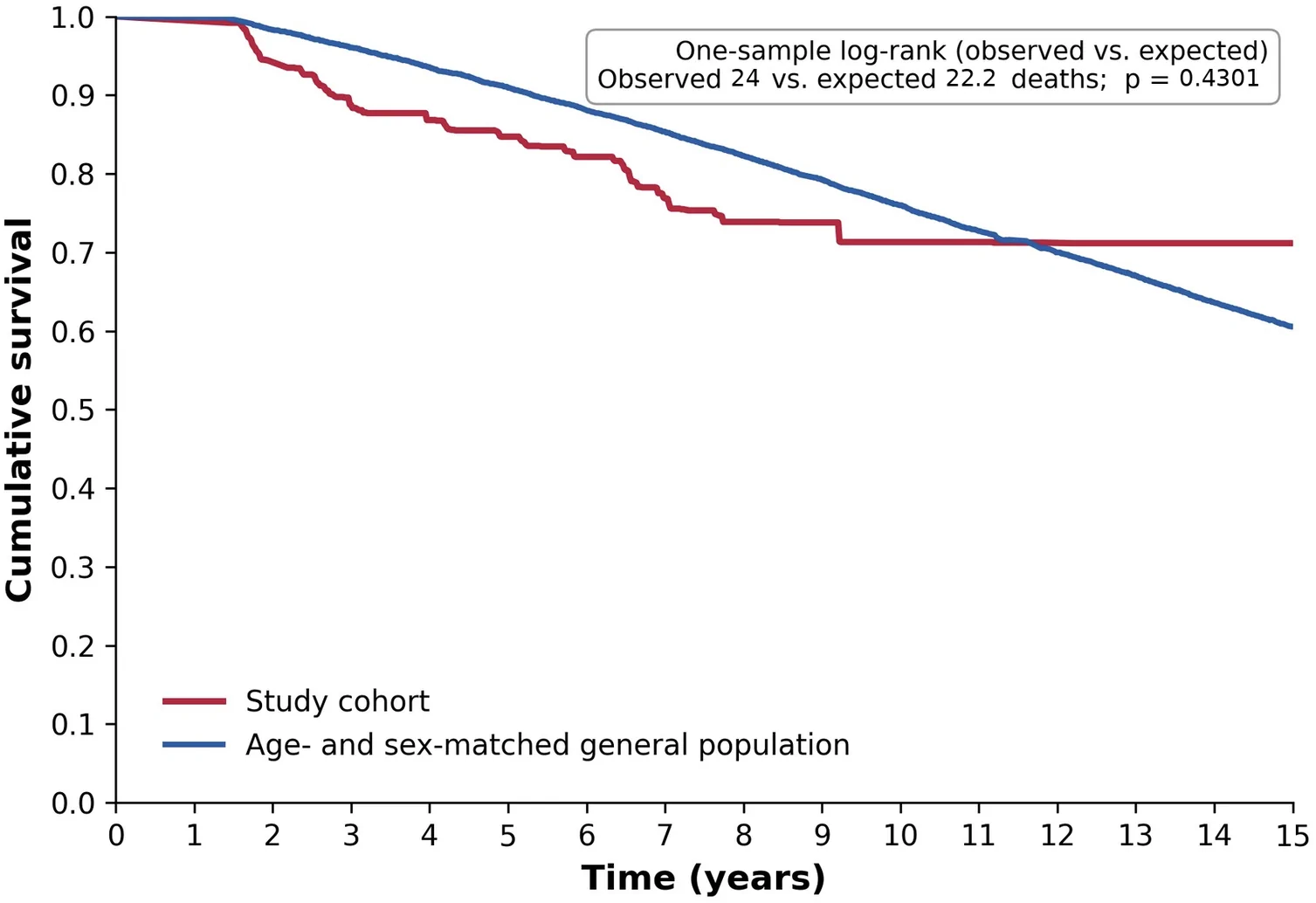
Survival comparison of the study cohort with an age- and sex-matched general population after exclusion of perioperative (three-month) mortality. Red line, study cohort; blue line, matched general population. One-sample log-rank test: observed 24 vs. expected 22.2 deaths; *p* = 0.4301.

Reoperation was required in 9 patients (8.0%): 2 for paravalvular leakage after replacement and 7 for recurrent mitral regurgitation after repair. Freedom from reoperation was 98.3% at 5 years and 93.3% at 8 years (Figure 4).

**Figure 4:**
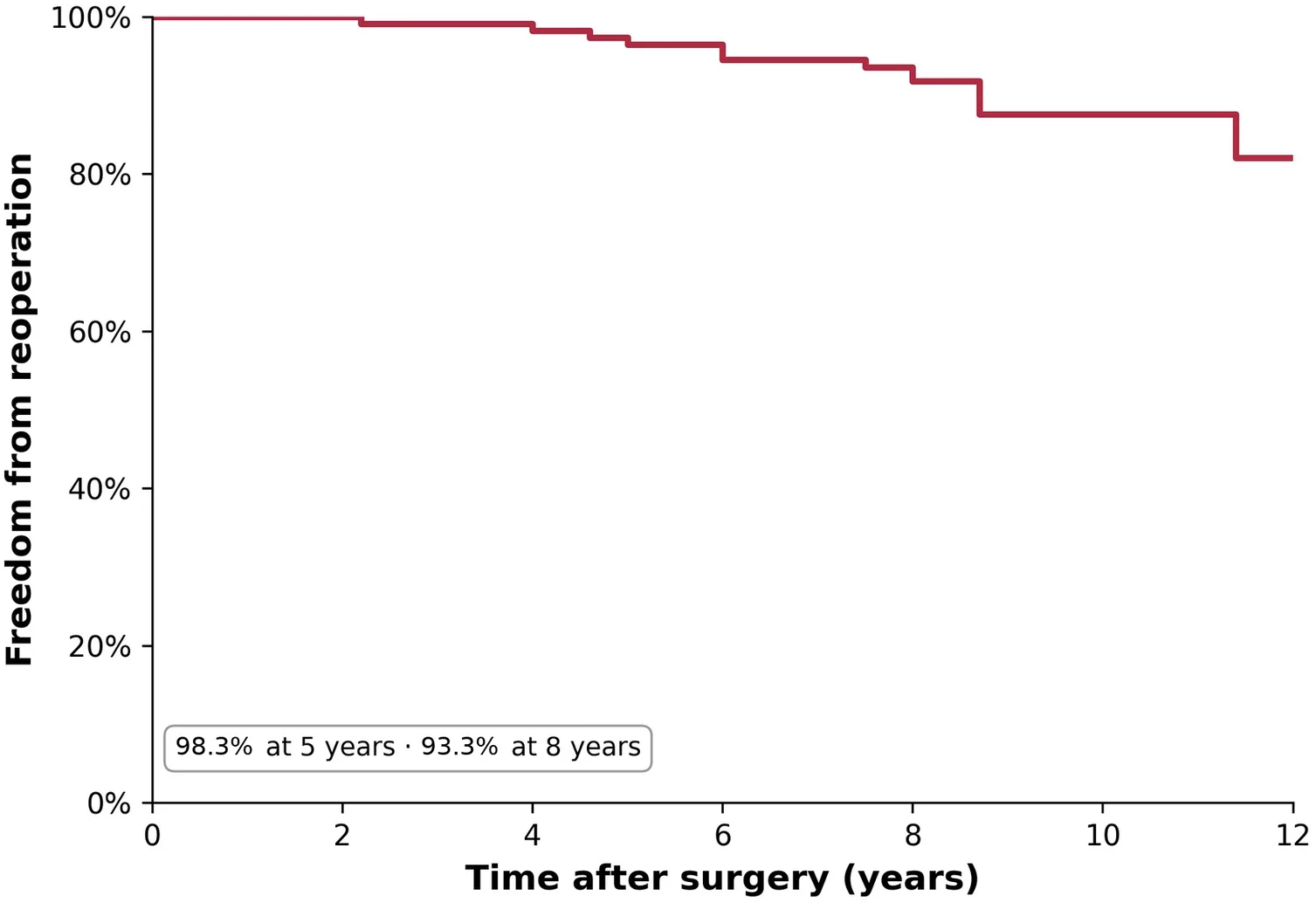
Freedom from reoperation over the postoperative course (98.3% at 5 years, 93.3% at 8 years).

## Discussion

MAC is associated with advanced age and female sex, with a reported female-to-male ratio of about 3:2 (2, 9). In this cohort more women than men died, both early (6 women, 1 man) and late (17 women, 9 men), and overall mortality was higher in women (69.7% vs 30.3%). The women were on average about a decade older than the men (70.3 vs 60.7 years), so age and sex could not be separated in an unadjusted analysis. Female sex, age and comorbidity have been reported as markers of worse outcome after mitral surgery in MAC (2, 9).

Age over 65 years was associated with higher early (*p* = 0.0323) and late mortality (*p* = 0.0155), as were arterial hypertension (*p* = 0.0003 and *p* = 0.001) and diabetes mellitus (*p* = 0.0050 and *p* = 0.0007). Previous cardiac decompensation was associated with both early (*p* = 0.0002) and late death (*p* = 0.0001) and was present in 35% of early deaths and 36% of late deaths but in only 3% of survivors. Chronic renal impairment was associated with higher late mortality (*p* = 0.0019). These associations are consistent with reported risk factors for mitral surgery in the setting of annular calcification (1, 10).

Valve repair was attempted whenever the valve allowed it and succeeded in 49% of patients; replacement was necessary in 51% because of the extent of calcification. Overall mortality was 36.8% after replacement and 21.8% after repair, with early mortality of 8.8% and 3.6% and late mortality of 28.1% and 18.2%, respectively. Patients who underwent replacement were older and more comorbid, so this difference reflects patient selection as well as the procedure. A preference for repair over replacement in degenerative mitral disease, where feasible, is supported by outcome data and current guidelines (11–16).

Concomitant procedures added considerable risk (17). The rate of additional procedures was 58%. Early mortality was higher after combined procedures (9.2%, 6 of 65) than after isolated mitral surgery (2.1%, 1 of 47), and concomitant procedures were associated with early mortality (*p* = 0.0382). Combined replacement and a further procedure was also associated with early death (*p* = 0.0062), as was additional aortic valve replacement (*p* = 0.0047), performed in 6 of the 7 early deaths. Additional coronary artery bypass grafting, carried out in 33 patients, was associated with higher overall mortality (*p* = 0.0232). Survivors had shorter operative (304 ± 81 vs 344 ± 79 min; *p* = 0.0339), bypass (188 ± 59 vs 211 ± 62 min; *p* = 0.0139) and cross-clamp times (121 ± 41 vs 145 ± 42 min; *p* = 0.0085) than non-survivors, and repair was consistently faster than replacement.

Reoperation was required in 8.0% of patients, most often for mitral regurgitation or paravalvular leakage. In survivors, echocardiography showed a competent valve in 47 patients, grade I regurgitation in 17, grade II in 4 and grade III in 3, with no grade IV. Left atrial diameter fell from 54 to 41 mm and LVEDD from 57 to 51 mm relative to baseline. Reported outcomes after annular decalcification and reconstruction in comparable cohorts are within a similar range (10, 18).

After the perioperative period, long-term survival was comparable to that of an age- and sex-matched general population (*p* = 0.4301 after exclusion of three-month mortality). With perioperative deaths included, observed mortality exceeded that expected in the matched population (*p* = 0.0016). Decalcification and patch reconstruction can restore a durable, well-functioning valve, but perioperative risk is high and should be considered in patient selection.

### Limitations

This retrospective single-centre study of 112 high-risk patients has some limitations. The associations between preoperative variables and mortality were derived from univariate analyses and should be regarded as associations rather than independent predictors, and residual confounding cannot be excluded. Long-term echocardiography was available for 72 of the 79 survivors, so the echocardiographic findings are subject to survivor bias. The comparison with the general population excluded perioperative mortality and represents conditional survival. Because concomitant procedures were performed almost exclusively through sternotomy and the minimally invasive subgroup was small, the surgical approaches could not be compared without confounding by procedure type. The cohort size, the near-complete follow-up and the mean follow-up of eight years nonetheless allow assessment of the long-term results of decalcification and patch reconstruction in this population.

## Conclusion

In this high-risk cohort, mitral valve surgery with annular decalcification and pericardial patch reconstruction provided durable valve function and favorable long-term outcomes among patients surviving the perioperative period. However, perioperative mortality remained substantial, underscoring the need for careful patient selection when considering annular decalcification and patch reconstruction for severe MAC.

## Data Availability

The original contributions presented in the study are included in the article/Supplementary Material, further inquiries can be directed to the corresponding author.
